# Structural and functional brain markers of cognitive impairment in healthcare workers following mild SARS-CoV-2 infection during the original stream

**DOI:** 10.1093/braincomms/fcae340

**Published:** 2024-09-30

**Authors:** Javier J González-Rosa, María P Gómez-Molinero, Elena Lozano-Soto, Silvia P Fernández-Rosa, Marina Campos-Silvo, María Paula García-Rodríguez, Fátima Cano-Cano, Florencia Sanmartino, Raúl Rashid-López, Paloma Macías-García, Jaime D Gómez-Ramírez, Raúl Espinosa-Rosso, José Paz-Espósito, Rocío Gómez-Molinero, Lucía Forero, Álvaro J Cruz-Gómez

**Affiliations:** Institute of Biomedical Research and Innovation of Cadiz (INiBICA), 11009 Cadiz, Spain; Psychology Department, University of Cadiz, 11510 Puerto Real, Spain; Radiodiagnostic Department, Jerez de la Frontera University Hospital, 11407 Jerez de la Frontera, Spain; Institute of Biomedical Research and Innovation of Cadiz (INiBICA), 11009 Cadiz, Spain; Psychology Department, University of Cadiz, 11510 Puerto Real, Spain; Radiodiagnostic Department, Jerez de la Frontera University Hospital, 11407 Jerez de la Frontera, Spain; Institute of Biomedical Research and Innovation of Cadiz (INiBICA), 11009 Cadiz, Spain; Radiodiagnostic Department, Jerez de la Frontera University Hospital, 11407 Jerez de la Frontera, Spain; Institute of Biomedical Research and Innovation of Cadiz (INiBICA), 11009 Cadiz, Spain; Institute of Biomedical Research and Innovation of Cadiz (INiBICA), 11009 Cadiz, Spain; Psychology Department, University of Cadiz, 11510 Puerto Real, Spain; Institute of Biomedical Research and Innovation of Cadiz (INiBICA), 11009 Cadiz, Spain; Neurology Department, Puerta del Mar University Hospital, 11009 Cadiz, Spain; Institute of Biomedical Research and Innovation of Cadiz (INiBICA), 11009 Cadiz, Spain; Psychology Department, University of Cadiz, 11510 Puerto Real, Spain; Institute of Biomedical Research and Innovation of Cadiz (INiBICA), 11009 Cadiz, Spain; Institute of Biomedical Research and Innovation of Cadiz (INiBICA), 11009 Cadiz, Spain; Neurology Department, Jerez de la Frontera University Hospital, 11407 Jerez de la Frontera, Spain; Radiodiagnostic Department, Puerta del Mar University Hospital, 11009 Cadiz, Spain; Psychology Department, University of Cadiz, 11510 Puerto Real, Spain; Institute of Biomedical Research and Innovation of Cadiz (INiBICA), 11009 Cadiz, Spain; Neurology Department, Puerta del Mar University Hospital, 11009 Cadiz, Spain; Institute of Biomedical Research and Innovation of Cadiz (INiBICA), 11009 Cadiz, Spain; Psychology Department, University of Cadiz, 11510 Puerto Real, Spain

**Keywords:** post-COVID-19 condition, cognitive deficits, structural MRI, functional connectivity, healthcare workers

## Abstract

Severe acute respiratory syndrome coronavirus 2 infection often involves the nervous system, leading to cognitive dysfunctions, fatigue and many other neurological signs that are becoming increasingly recognized. Despite mild forms of the disease accounting for most cases worldwide, research on the pathophysiology driving mild coronavirus disease 2019 (COVID-19) has received little attention. In this respect, recent evidence has pointed out that around 30–40% of non-critical, mild-to-moderate severity COVID-19 survivors may display cognitive disturbances several months post-illness. Hence, the impact of COVID-19 on the brain structure and function, through potential neuropathological mechanisms underpinning cognitive alterations in post-mild COVID-19 infections, remains largely unexplored. This retrospective multicentre observational cohort study, entirely based on a healthcare worker sample (*n* = 65; 55% females, aged 21–61), investigated the cognitive status and the structural and functional brain integrity among non-hospitalized individuals who developed mild COVID-19 symptoms during the occurrence of severe acute respiratory syndrome coronavirus 2 variants Alpha to Delta, compared with healthy controls tested before the pandemic onset. All evaluations were performed at an average of 9-month follow-up post-infection period. Participants completed a comprehensive neuropsychological assessment and structural and functional MRI exams. Radiological inspection sought to detect the presence of white matter hyperintensities on axial fluid-attenuated inversion recovery images. Global and regional grey matter integrity assessment, analysing changes in grey matter volumes and cortical thinning, and functional connectivity alterations of resting-state brain networks were also conducted. Regression analyses tested the relationships between the presence of specific cognitive impairments and potential structural and functional brain findings. Our results revealed that clinical, cognitive screening and neuropsychological examinations were average between both groups, except for specific impairments related to executive functions in the mild COVID-19. Compared to healthy controls, mild COVID-19 subjects exhibited increased juxtacortical white matter hyperintensities, thalamic and occipital volume loss and diminished resting-state functional connectivity involving the left precuneus and cuneus in default-mode network and affecting the right angular gyrus and left precuneus in the dorsal attentional network. Reduced thalamic volume was the only variable selected in the final model explaining the observed executive function impairment in mild COVID-19. The presence of cognitive, structural and functional brain abnormalities over time suggests that the action of widespread neurovascular and inflammatory phenomena on the nervous system might also occur in mild forms following COVID-19 infection rather than permanent brain damage linked to the direct or indirect action of the virus. Our findings emphasize the need to pay attention to the long-term brain-related consequences of mild COVID-19 infections during the original stream.

## Teaser

Gonzalez-Rosa *et al*. report that healthcare workers, even following mild SARS-CoV-2 infection during the original stream, may develop executive function deficits, increased juxtacortical white matter hyperintensities, thalamic and occipital volume loss and diminished resting-state functional connectivity in posterior brain areas. Furthermore, cognitive deficits were associated with reduced thalamic volume.

## Graphical Abstract

**Graphical Abstract:**
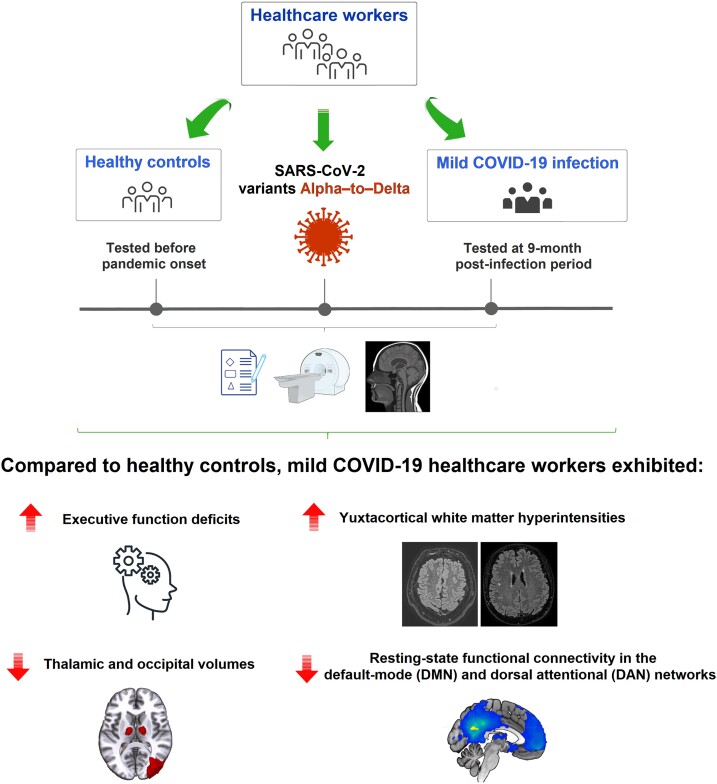

## Introduction

Severe acute respiratory syndrome coronavirus 2 (SARS-CoV-2) is an infectious and highly contagious disease that was officially identified in December 2019 as the cause of a respiratory illness designated coronavirus disease 2019 (COVID-19), which soon became a public health emergency with more than 600 million of confirmed cases worldwide and substantial mortality rates.^1^

Beyond affecting the respiratory system, SARS-CoV-2 can also cause devastating effects in multiple organ systems, including the central nervous system^2^ and, hence, a broad spectrum of neurological symptoms with high incidence^2-5^. Furthermore, neurological sequelae may persist, or even appear months after infection, as a part of a clinical condition called ‘post-COVID-19 syndrome’ (PCS),^6^ which suggests the presence of long-term effects of SARS-CoV-2 in the nervous system.^7^ Severe fatigue, psychiatric disorders and cognitive impairment are among the most reported sequelae in PCS^8-11^. According to the latest findings, cognitive sequelae in PCS patients are associated with dysfunctional neurological signalling that is also paired with a cascade of pathological changes in blood and cerebrospinal fluid biomarkers linked to neural and axonal damage as well as astrocyte and myelin changes that have been present since the acute phase of the disease^4,12-15^.

Although neurological sequelae might be more significant in patients with severe forms of COVID-19,^16^ patients with mild COVID-19 clinical forms remain widely unexplored and underrepresented in literature, as it has been recently highlighted.^8^ Moreover, these symptoms may remain undetected in several PCS and mild-to-moderate COVID-19 patients due to its subtle and progressive clinical manifestation over time or misattribution to causes other than COVID-19.^17^

Nonetheless, the problem of radiological and cognitive abnormalities in mild COVID-19 is open, as many questions remain unanswered. Surprisingly, even though healthcare workers have higher infection rates and are more likely to be exposed to this infection than the general population,^18,19^ few studies have reported mild COVID-19 sequelae in this particular segment of the population.^20^ Of note, several reports have demonstrated in healthcare workers the existence of long-term psychological complications such as depression, anxiety, insomnia and cognitive dysfunction,^20-22^ which, in turn, have a detrimental effect on job performance and psychosocial well-being.^23,24^ Regarding cognitive deficits, studies using techniques such as magnetic resonance imaging (MRI) have demonstrated brain morphological and functional changes due to SARS-CoV-2 infection, including the presence of inflammation, hyperintensities, hypoperfusion and cellular damage,^7,25^ highlighting noteworthy associations between radiological and cognitive measures.^15,26^ Interestingly, a recent longitudinal study found grey matter (GM) volume reductions in several brain regions in PCS patients 11 months after COVID-19’s first symptoms, even though only 4% of case patients assessed had been hospitalized.^27^

As the COVID-19 pandemic continues, further studies are necessary to determine the presence of changes in brain architecture and their relationship with cognitive symptoms in mild COVID-19 cases, in which symptoms and radiological findings such as white matter (WM) hyperintensities (WMHs) and GM reductions and functional connectivity changes are more likely to remain undetected. Here, the main goal of this retrospective multicentre observational cohort study was to explore the presence of short- and middle-term cognitive outcomes, the frequency and topography of cerebral WMHs and regional changes in the GM volume and cortical thickness, as well as the integrity of functional networks during the resting state among healthcare workers in mild forms of COVID-19 infections during the original strain compared to healthy individuals evaluated before the onset of the COVID-19 pandemic.

## Materials and methods

### Design and participants

Sixty-five participants were initially recruited as healthy volunteers as part of a multicentre observational MRI and neuropsychological follow-up study conducted between January 2019 and December 2021 that aimed to investigate cross-sectionally and longitudinally how cognitive and brain structural changes interrelate across the adult lifespan in neurological patients and healthy population. Participants were engaged via the Internet, poster advertising and hospital communications campaigns in two hospital centres, where all assessment and testing were conducted. A total of 17 individuals were excluded from this study for not completing all assessments (*n* = 3), having corrupted MRI data (*n* = 4), displaying contraindications to MRI (*n* = 3) or having doubts about meeting the mild severity COVID-19 diagnosis criteria (*n* = 8). The final sample was composed of 48 healthcare workers who met the inclusion criteria and were divided into two groups: the COVID-19 and the healthy control (HC) group.

The COVID-19 sample consisted of 24 individuals with confirmed infections during the period of SARS-CoV-2 variants Alpha to Delta,^28^ who were enrolled in this cross-sectional study in the mild COVID-19 group based on the following inclusion criteria: (i) age between 18 and 65 years; (ii) confirmed SARS-CoV-2 positivity in electronic medical records both on RT-PCR from the nasopharyngeal swab, antigen test or serology, including self-reported testing positive for SARS-CoV-2 (diagnosis from March 2020 to January 2021); and (iii) non-critical, mild SARS-CoV-2 infection along with asymptomatic cases. The exclusion criteria were (i) required hospitalization or admission to intensive care units due to COVID-19 complications; (ii) previous manifestation of moderate-to-severe COVID-19 symptoms, such as shortness of breath, dyspnoea or abnormal chest imaging, moderate or severe fatigue, neurological sequelae or confirmed PCS; (iii) presence of neurological, psychiatric or severe medical conditions; and (iv) contraindications for MRI assessment. After our study assessment, most COVID-19 participants were also infected with other circulating virus variations, resulting in additional symptoms and clinical severity, making the longitudinal assessment of this group impractical.

In addition, 24 participants conveniently matched in gender, age and educational level with no history of confirmed SARS-CoV-2 infection and assessed before the COVID-19 pandemic (before February 2020), and no other neurological, psychiatric or medical illnesses were selected to conform the HC group. All participants were healthcare workers at the Institute of Biomedical Research and Innovation of Cadiz, the Puerta del Mar University Hospital and the Jerez de la Frontera University Hospital in Cadiz, Spain, and included doctors, health researchers, nurses and paramedical and ancillary staff. The protocol for the study was approved by the Andalusian Biomedical Research Ethics Committee (Ref.: LFD-VIT-2018-01; EFCOEN3/PEIBA 1687-N21). All participants gave informed written consent before participation.

### Clinical and neuropsychological assessment

Participants were administered a comprehensive battery of neuropsychological assessments encompassing 12 sub-scores and assessing information processing speed, attention, working memory, episodic memory and executive functions, including a general screening test. Functional and medical information was also collected, and all standardized neuropsychological and neuropsychiatric tests were validated for the Spanish population. Neuropsychological data were gathered in the 2 weeks preceding or following the MRI assessment and included Mini-Mental State Examination^29^; Symbol Digit Modalities Test^30^; Paced Auditory Serial Addition Test (PASAT)^31^; Selective Reminding Test (SRT),^32^ including long-term storage consistent long-term retrieval and delayed recall sub-scores; 10/36 Spatial Recall Test,^33^ including long-term storage and delayed recall sub-scores; Phonemic Verbal Fluency and Semantic Verbal Fluency^34^ tests; and Digit Span Subtest of the Wechsler Adult Intelligence Scale,^35^ including both digits forward and digits backward (DB). In addition, psychiatric, fatigue and quality of life measures were also obtained during the neuropsychological assessment, including the Beck Depression Inventory-second edition,^36^ State-Trait Anxiety Inventory,^37^ Fatigue Severity Scale^38^ and World Health Organization Quality-of-Life Scale-Brief Version.^39^

### Neuroimaging

#### Imaging acquisition

All participants underwent an MRI scan using a 1.5 T scanner, in which both 3D T1-weighted and axial fluid-attenuated inversion recovery (FLAIR) images and a resting-state functional MRI sequence were acquired. The following sequences were collected: (i) T1-weighted magnetization-prepared rapid acquisition gradient-echo 3D sequence (TR/TE = 7.27/3.32 ms; flip angle = 15°; matrix = 180 × 288 × 288; voxel size = 1 × 0.9 × 0.9 mm; 180 slices); (ii) FLAIR 3D sequence (TR/TE = 6000/354 ms; flip angle = 180°; matrix = 180 × 256 × 256; voxel size = 1 × 1 × 1 mm; 180 slices); and (iii) open-eyes resting-state fMRI images by a gradient echo-planar imaging sequence of 150 volumes (TR/TE = 3000/47 ms; flip angle = 90°; matrix = 96 × 96 × 36; voxel size = 2.6 × 2.6 × 3.5; 36 slices; slice order acquisition = ascending).

#### Radiological analysis

Two experienced radiologists blinded to clinical data performed visual assessments of WMHs based on 3D-FLAIR images. Although WMHs are mainly located in the periventricular WM and perivascular spaces, they can also be detected in deep and juxtacortical WM.^40,41^ WMHs were classified as being or involving cortical, subcortical, juxtacortical, periventricular, centrum semiovale, basal ganglia, corona radiata, internal capsule, brainstem, cerebellar peduncles and corpus callosum sites. The radiologists rated independently and randomly the same 48 scans using the quantitative visual rating Fazekas and Schmidt scale.^40,41^ The frequencies and percentages of WMHs in each group and brain localization were reported.

#### Neuroimaging structural analysis

First, 3D T1 images were visually inspected for possible artefacts and then preprocessed using the Computational Anatomy Toolbox (CAT12, version 12.6 r1450, https://neuro-jena.github.io/cat//,^42^ an extension to Statistical Parametrical Mapping (SPM12, Wellcome Department of Cognitive Neurology, https://www.fil.ion.ucl.ac.uk/spm/). The preprocessing steps included (i) reorientation to the anterior–posterior commissure; (ii) bias-field correction; (iii) segmentation into the GM, WM and cerebrospinal fluid; (iv) spatial normalization of GM images to the montreal neurological institute (MNI) space; and (v) modulation of the normalized data by scaling the voxel values to compensate volume changes caused by non-linear registration during spatial normalization. GM volumes of interest of 170 areas were extracted using the third version of the Anatomical Automatic Labeling (AALv3) atlas^43^ and corrected for brain size using total intracranial volume as a covariate in subsequent statistical design.

In addition, global measures of brain atrophy were brought. Specifically, brain parenchymal fraction and GM fraction were obtained,^44^ and to explore regional differences in GM volumes between groups, region of interest (ROI) analysis was performed using CAT12. Cortical thickness measures were also addressed through CAT12, following a fully automated method that uses tissue segmentation to estimate distances between WM and GM voxels based on the projection-based thickness method.^45^ Regional cortical thickness values were extracted using the Desikan–Killiany atlas.^46^

#### Neuroimaging resting-state functional connectivity analysis

The preprocessing of resting-state functional magnetic resonance imaging data was performed with the toolbox Data Processing & Analysis for Brain Imaging.^47^ We followed the default preprocessing pipeline, which included the following steps: (i) deleting the first five time points to allow the magnetization to approach a dynamic equilibrium; (ii) slice timing correction for temporal alignment; (iii) realignment to first scan to correct the subject head motion; (iv) co-register to high-resolution 3D T1 anatomical image of reference; (v) segmentation of GM, WM and cerebrospinal fluid maps; (vi) normalization to the MNI space; and (vii) smoothing of 4 mm full width half maximum (FWHM) Gaussian kernel. Two participants (one for each group) were excluded from these analyses for excessive head motion during resting-state fMRI data acquisition (defined as head movement greater than 1 mm/1° across six translation and rotation axes during the functional recording). Subsequently, an independent component analysis (ICA) was performed, including the estimation and extraction of components following the infomax algorithm and dual regression back-reconstruction, performed with the toolbox Group ICA of fMRI Toolbox software.^48^ Specific spatial maps of the two most consistent resting-state networks reported in the literature and widely related to cognition were previously identified and selected for subsequent voxel-wise statistical analyses: default-mode network (DMN) and dorsal attentional network (DAN).^49^

## Statistical analysis

SPSS 25 (IBM, Armonk, NY) and custom SPSS syntax routines were used to analyse clinical, neuropsychological and neuroimaging global structural measures. After verifying the normal distribution of all continuous variables (Shapiro–Wilk test), differences between groups were performed using Student’s *t*-test for continuous variables and chi-square (*χ^2^*) for categorical variables. To control for age and educational level effects in clinical, psychiatric and neuropsychological variables, a general linear model univariate procedure was performed for each dependent variable, including group as fixed factor (HC/COVID-19), and age and years of education as covariates. Regarding GM and cortical thickness ROI analyses, significant differences between groups were assessed using the Analyze ROIs function included in the CAT12 software, following a two-sample *t*-test model and adopting the Holm–Bonferroni correction for multiple comparisons (*P* < 0.05). Voxel-wise statistical differences between groups in ICA were assessed with the SPM12 statistical module, following the general linear model. First, DMN and DAN individual functional connectivity maps were entered in a one-sample test design in SPM12 to obtain a global mask of each network, which subsequently assessed significant differences between groups using a two-sample *t*-test design. Both designs were presented at *P* < 0.05 family-wise error (FWE) cluster-level correction for multiple comparisons in combination with a threshold of *P* < 0.001 at the voxel level.

Additionally, Pearson’s partial correlation coeﬃcient (*ρ*), adjusted for age and educational level, was calculated separately to screen clinical, regional GM volume and resting-state network functional connectivity variables for their relationship with the impaired cognitive tests only for the COVID-19 group. First, only those variables previously showing statistical significance or a trend towards statistical significance between groups were further selected and explored. Second, only those variables related to clinical, GM volume and resting-state functional connectivity that showed significant partial correlations, controlling age and educational level and after false discovery rate (FDR) adjustment (*P* < 0.05) for multiple testing, were then entered in a multiple linear regression model with simultaneous method (enter) to determine the most robust clinical and MRI predictors of cognitive impairment.

A different linear regression model was generated for each impaired cognitive domain (i.e. DB, phonemic fluency and PASAT-3). The residuals obtained from the partial correlations (after adjusting for age and educational level for each significant variable) were first saved and then entered as new clinical and neuroimaging variables into the simultaneous linear regression model (entrance criterion *P* < 0.05 and exit criterion *P* = 0.10) as predictors of each impaired cognitive test score.

## Results

### Prevalence of impaired cognitive functioning

Participant’s main demographical, psychiatric and neuropsychological characteristics are summarized in Table 1. COVID-19 and HC did not significantly differ in age, educational level and gender proportion. No significant differences between groups were found regarding fatigue severity levels and psychiatric scores, while COVID-19 showed a statistical trend towards higher levels of state anxiety and depression. Although no significant differences were observed between groups in Mini-Mental State Examination total score and most cognitive subscales, after adjustment for age and educational level, COVID-19 showed statistically significant reduced scores in DB and phonetic fluency neuropsychological tests compared to HC, while a statistical trend towards impairment was also observed in PASAT-3 scores (Table 1). Cognitive impairment was interpreted as a failure on two or more neuropsychological tests, while test failure was determined as scoring lower than 1.5 standard deviation from the average of the HC group. Following this criteria, nine COVID-19 subjects (36%) were classified as cognitively impaired. The aforementioned cutoff criterion has been suggested as the optimal threshold for identifying abnormal performance on a neuropsychological assessment and subsequently recommended for the categorization of cognitive impairment severity^50-52^.

**Table 1:** Clinical and neuropsychological data, global MRI measures, and radiological findings

	HC (*n* = 24)	COVID-19 (*n* = 24)	*P*-values (*F*, *t*, *χ*^2^)
**Demographic data**			
Age	39.67 (10.24)	45.17 (10.66)	0.075^a^
Educational level (years)	17.33 (2.81)	16.13 (2.56)	0.126
Sex (female/male)	16/8	15/9	0.763
Time between COVID-19 onset and neuropsychological assessment/MRI (months)		9.03 (5.55)	
**Quality of life**			
WHOQOL-BREF	99.08 (15.42)	100.43 (13.84)	0.645
**Fatigue severity**			
FSS	27.46 (12.59)	32.58 (14.99)	0.278
**Depression**			
BDI-II	7.63 (6.85)	13.92 (15.02)	0.085^a^
**Anxiety**			
STAI-State	19.63 (10.95)	14.63 (8.45)	0.078^a^
STAI-Trait	19.08 (11.11)	18.92 (12.31)	0.968
**Cognition**			
MMSE	29.88	29.79	0.742
DF (WAIS-III)	6.33 (1.20)	6.04 (1.63)	0.915
DB (WAIS-III)	5.33 (1.09)	4.58 (0.97)	**0.013**
SDMT	61.75 (13.91)	58.92 (10.97)	0.801
PASAT-3sec.	50.38 (8.01)	44.50 (10.75)	0.091^a^
SRT long-term storage	54.92 (9.86)	54.42 (13.24)	0.587
SRT consistent long-term retrieval	48.42 (13.87)	47.21 (16.74)	0.491
SRT delayed recall	9.63 (2.39)	10.08 (1.66)	0.136
10/36 SPART long-term storage	22.29 (3.77)	21.54 (4.64)	0.512
10/36 SPART delayed recall	7.83 (1.93)	7.33 (2.10)	0.374
Phonemic fluency (F)	14.54 (3.50)	11.96 (3.82)	**0.016**
Semantic fluency (animals)	24.29 (4.96)	24.83 (5.16)	0.625
**MRI global measures**			
BPF	0.82 (0.03)	0.82 (0.03)	0.488
GMF	0.46 (0.02)	0.45 (0.02)	0.314
Global cortical thickness (mm)	2.46 (0.11)	2.45 (0,07)	0.707
**WMH detection sites** (percentages of participants in each category)			
Cortical			
Subcortical	31.42	35.48	0.450
Juxtacortical	5.71	37.50	**0.020**
Periventricular	4.16	8.33	0.652
Centrum semiovale	6.45	11.42	0.132
Basal ganglia	4.16	4.16	0.999
Corona radiata	4.16	8.33	0.652
Internal capsule	11.12	12.50	0.852
Brainstem			
Corpus callosum			
Cerebellar peduncles			

Means and standard deviations for each group are presented for continuous variables and frequency values for categorical variable (sex). The between-group comparison of quality of life, fatigue severity, depression and anxiety variables was corrected for age, while the between-group comparison of cognition variables was corrected for age and educational level.

***Abbreviations*:** BDI-II = Beck Depression Inventory-II; BPF = brain parenchymal fraction; COVID-19 = coronavirus disease 2019; DB = digits backward; DF = digits forward; FSS = Fatigue Severity Scale; GMF = grey matter fraction; HC = healthy control; MMSE = Mini-Mental State Examination; MRI = magnetic resonance imaging; PASAT = Paced Auditory Serial Addition Test; SDMT = Symbol Digit Modalities Test; SPART = Spatial Recall Test; SRT = Selective Reminding Test; STAI = State-Trait Anxiety Inventory; WAIS = Wechsler Adult Intelligence Scale; WM: white matter; WHOQOL-BREF = World Health Organization Quality-of-Life Scale-Brief Version.

^a^Indicates a statistical trend and bold font indicates statistical significance (*P* < 0.05) between groups.

### WMH findings and global MRI outcomes

**Figure 1:**
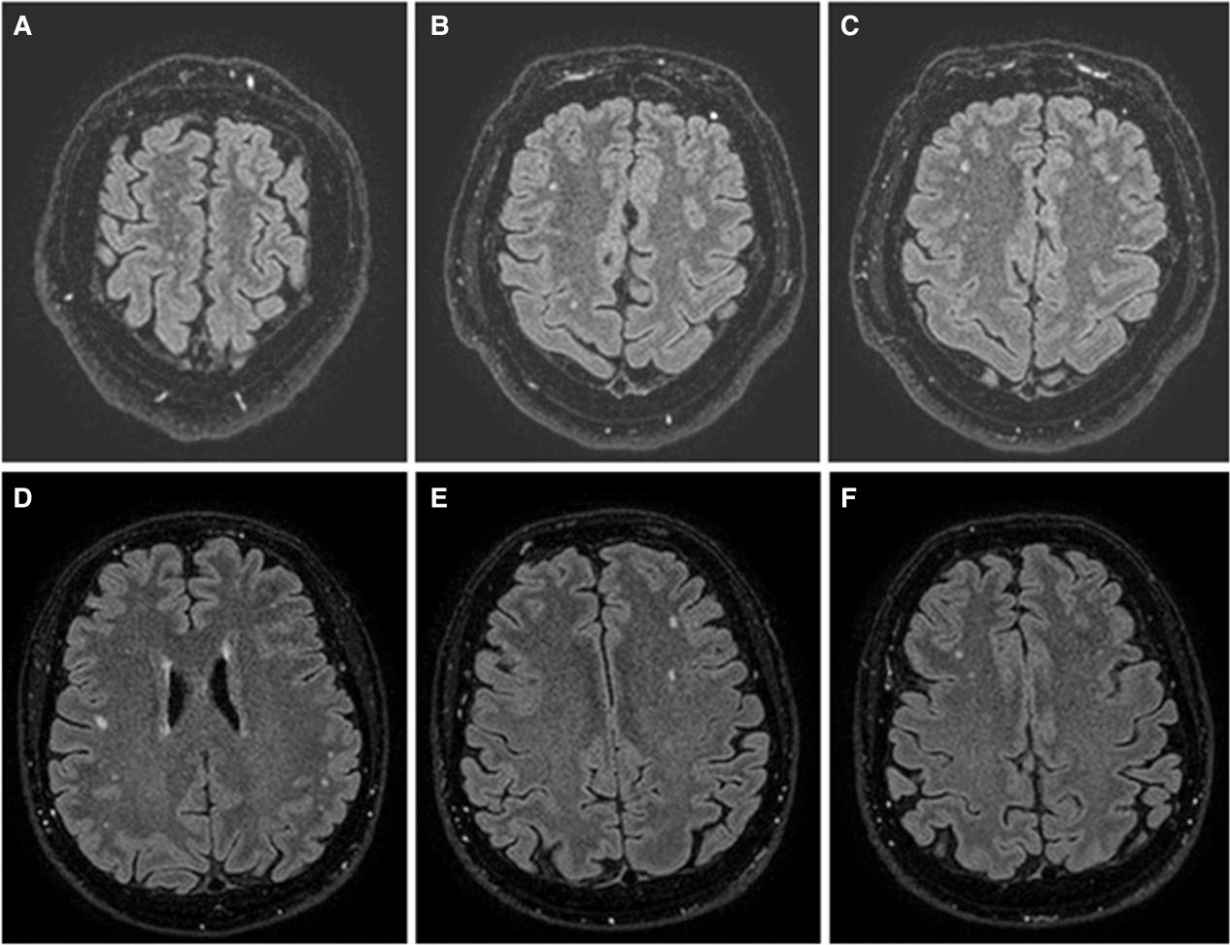
**FLAIR images in two different representative participants with mild COVID-19.** FLAIR images 9 and 4 months after mild COVID-19 symptom onset, respectively, (**A–C**) in a 46-year-old man depicting multiple nonconfluent multifocal WMHs on FLAIR images, rounded/ovoid shape, mostly located in the juxtacortical frontoparietal bilateral WH, without any mass effect nor diffusion restriction and (**D–F**) in a 61-year-old man showing similar findings to the previous, adding some other locations for the hyperintensity on FLAIR images, such as the left centrum semiovale.

Regarding MRI global measures, no significant differences between groups were found in BPF, GMF and global cortical thickness. As for the distribution of WMHs by the brain ROI, most of the participants in both COVID-19 and HC groups exhibited WMHs over subcortical WM (35.48 versus 31.42%, respectively), followed by the presence of WMHs in the centrum semiovale (11.42 versus 6.45%) and the internal capsule (12.50 versus 11.12%). Remarkably, our results also revealed a statistically significant increase (*P* = 0.020) of juxtacortical WMHs in mild COVID-19 (37.50%) compared to HC (5.71%) (Table 1) (Fig. 1).

### Regional GM volume and cortical thickness

**Figure 2:**
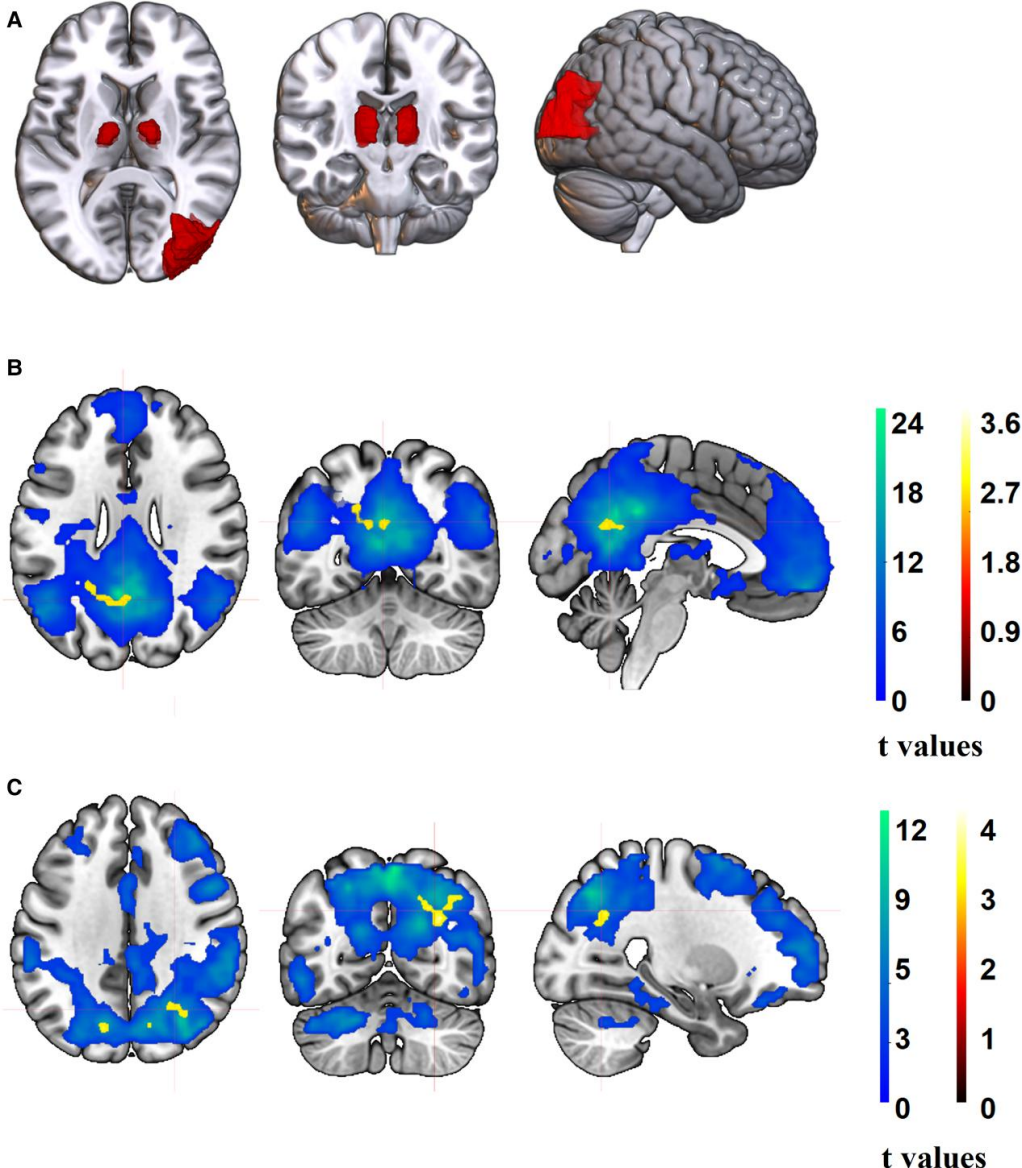
**Significant group differences in regional GM volumes and areas of resting-state functional connectivity between mild COVID-19 and HCs.** (**A**) ROI analysis showing significant GM reductions (in red) in recovered COVID-19 healthcare workers (*n* = 24) compared to HCs (*n* = 24) in bilateral TVL nuclei and right middle occipital gyrus (MOG) (*P* < 0.05, Holm–Bonferroni multiple comparison corrected). (**B**) Resting-state ICA analysis showing one sample *t*-test for DMN in the whole sample (blue) and a significant cluster (yellow) of reduced functional connectivity involving the left precuneus and cuneus in COVID-19 healthcare workers (*n* = 23) compared to HC (*n* = 23) (FWEc = 36, *P* < 0.001). (**C**) Resting-state ICA analysis showing one sample *t*-test for the DAN in the whole sample (blue) and significant clusters (yellow) of reduced functional connectivity involving the right angular gyrus and precuneus and left precuneus and cuneus in COVID-19 compared to HC (FWEc = 23, *P* < 0.001).

ROI-based analysis showed a significant GM volume reduction in mild COVID-19 affecting bilateral thalamic ventral lateral (TVL) nuclei and in the right middle occipital gyrus (MOG) (*P* < 0.05, Holm–Bonferroni corrected) (Fig. 2A). Opposite contrast did not show any significant results. Of note, cortical thickness regional measures showed no significant differences between groups.

### Patterns of resting-state functional connectivity

Our results revealed a pattern of resting-state hypoconnectivity in both DMN and DAN in mild COVID-19 versus HC. More specifically, COVID-19 exhibited significantly weaker functional connectivity in a cluster (36 voxels) within the DMN involving the left precuneus and cuneus (*P* < 0.0001 at voxel level with FWE cluster correction) (Fig. 2B) and in two clusters (53 and 29 voxels) within the DAN involving the right angular gyrus and left precuneus, respectively (*P* < 0.001 at voxel level with FWE cluster correction) (Fig. 2C). Our findings did not reveal significant increases in resting-state functional connectivity in COVID-19 compared to HC.

### Clinical and neuroimaging predictors of cognitive impairment in mild COVID-19

**Figure 3:**
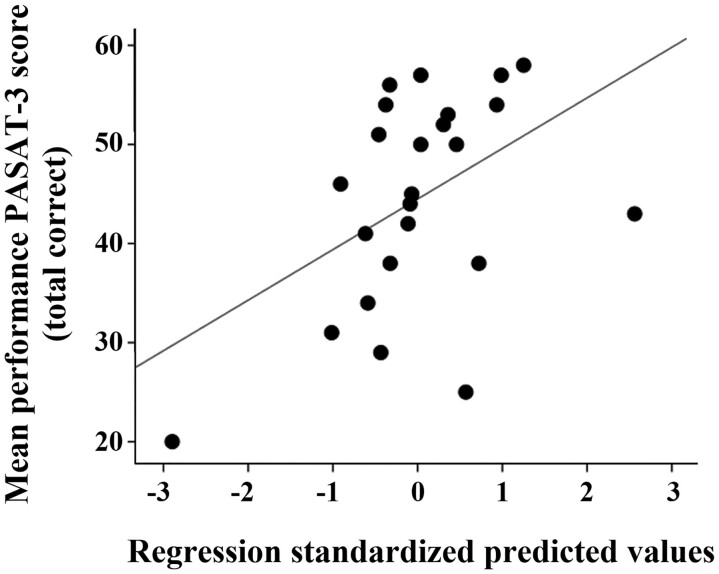
**Associations between regression standardized predicted values and PASAT-3 scores in the mild COVID-19 group**. Linear stepwise regression model retaining GM volume of the right TVL nucleus (*β* = −0.475, *t* = −2.529) as significant predictors of PASAT-3 performance (adjusted for age and educational level; *R*^2^ = 0.225, *P* = 0.019). The *y*-axis represents the PASAT-3 scores, and the *x*-axis represents the averaged residuals of age and educational level of the GM volume in the right TVL. Each data point represents a COVID-19 participant.

To further describe the relative association between the impaired neuropsychological test and regional neuroimaging measures (anatomical regional parcellations for GM atrophy and functional connectivity network nodes), individual tests were initially assessed using partial correlations controlling for age and educational level and multiple testing adjustment. None of the considered neuroimaging predictors were associated with impaired DB and phonetic fluency. However, only atrophy of the right TVL nucleus remained significant in the final model, explaining the reduced cognitive performance of the mild COVID-19 in the PASAT-3 (*R*^2^ = 0.225, *P* = 0.019) (Fig. 3).

## Discussion

The findings of this study underlined that non-hospitalized healthcare workers who had mild COVID-19 during the first surges involving Alpha to Delta sublineages of SARS-CoV-2, with no specific neurological manifestations, and compared to an HC group assessed before the onset of the pandemic, exhibited both subclinical cognitive impairments and altered structural and functional brain integrity at 9-month follow-up.

The reduced neuropsychological performance displayed by the COVID-19 group was closely linked to cognitive domains involving working memory, information processing speed, phonemic verbal fluency and executive functions, which appears to be, furthermore, independent of the presence of mood symptoms or fatigue. Several reports have previously demonstrated the presence of cognitive dysfunction in patients with past COVID-19 infection, highlighting particularly the relevance of executive functioning impairments^10,53-58^. Importantly, although some studies suggest a positive association between cognitive impairment and disease severity, wherein cognitive deficits seem to be more prominent in hospitalized individuals,^54,59^ recent evidence suggests that these deficits could also be noted in outpatient individuals,^55^ moderate COVID-19 cases^58,60^ or asymptomatic patients.^61^ Furthermore, this sustained subclinical cognitive impairment in healthcare workers with COVID-19 could develop subtly, regardless of the sub-acute or post-acute phase of evaluation,^62^ clinical course or severity of the COVID-19 infection.^56,57,62^ Consequently, this condition is increasingly recognized as a common complication after recovering from mild COVID-19.^53,58,63,64^

Interestedly, despite the healthcare workers with COVID-19 of our study not indicating subjective cognitive complaints, most of them displayed reduced neuropsychological performance, which is in line with recent reports demonstrating the presence of unnoticed cognitive deficits even in individuals who apparently ‘fully’ recovered from a mild form of COVID-19 without expressing subjective complaints.^65^ These results point out that the sole administration of self-reported cognitive assessments and cognitive screening tests could underestimate the presence of cognitive dysfunction,^66^ which could be particularly relevant in the heatlhcare worker population. Furthermore, the presence and impact of observed cognitive abnormalities in this group remained a few months following the infection, despite current evidence that seems to indicate that there would not be a persistent cognitive impairment caused by mild infection.^67^ Moreover, the reduction in neuropsychological scores on executive tasks found among mild COVID-19 could be linked to the hypoconnectivity observed within the DAN and DMN networks. This observation follows previous reports demonstrating altered functional connectivity during a few-month follow-up in moderate-to-severe recovered COVID-19 patients.^68,69^ Nevertheless, these results suggest that the heterogeneous cognitive and clinical manifestations of PCS seem to be not associated with specific resting-state fMRI findings, indicating a loss of specificity for specialized resting-state networks several months after mild COVID-19 infection.

Remarkably, our study also revealed no signs of brain atrophy or GM volume abnormalities at the global level, nor any alterations associated with global or regional thinning in cortical regions in mild COVID-19. Nevertheless, the regional volumetric analysis revealed reduced bilateral thalamic volumes (concretely within the TVL in mild COVID-19 cases, comparable to the middle occipital gyrus). To our understanding, no studies have reported occipital GM loss in patients with mild COVID-19. Generally, the GM atrophy in the occipital areas may be a secondary consequence caused by the disrupted long-association fibres induced by vascular lesions,^70^ as the occipital regions are the main origin and destination of many of these fibres.^71^ We speculate that the pathophysiological mechanisms behind structural changes in the middle occipital gyrus observed in mild COVID-19 might be the result of the widespread brain abnormalities and disruption of functional intrinsic brain activity and disease-related adaptations in sensory cortices, which could be linked to the functional connectivity alterations in visual processing-related areas also observed in mild COVID-19. In fact, our results showed that disrupted resting-sate network connection patterns can impact DMN and DAN and involve occipital network-related regions. The precuneus and cuneus have been identified as indispensable areas in DMN and DAN^72-74^ and are also known to be preferentially involved in visual information processing and attentional deployment. The reduced functional connectivity of these visual processing-related networks may be one of the reasons for increased attention and fatigue-related deficits commonly observed among COVID-19 survivors. However, we did not find a significant association between precuneus/cuneus/visual area connectivity of the DMD–DAN networks and cognitive performance, which may be due to the limited sample size or the severity of cognitive deficits observed in the mild COVID-19 group; hence, the mechanism of functional connectivity changes in posterior brain networks after COVID-19 remains to be determined. Nevertheless, increased inflammatory response following acute SARS-CoV-2 infection may cause dysconnectivity in several resting-state fMRI networks,^75^ which could facilitate the emergence of cognitive and mood symptoms among these patients^76^ and eventually make some of them more vulnerable to the presence of these PCS manifestations.

Volumetric and functional abnormalities affecting the thalamus have been previously recognized in COVID-19 patients, including ‘long’ COVID or PCS.^27,77^ Generally, thalamic volume loss is hypothesized to result from axonal degeneration secondary to white matter injury. Thalamic degeneration is usually prominent due to its extensive connections between subcortical areas and the neocortex through a large number of WM tracts, which include both the motor and visual cortex.^78^ Particularly, the ventral lateral nucleus of the thalamus receives inputs from the cerebellum and basal ganglia and relay cerebellar signals to the primary motor area, playing a critical role in fine motor control.^78^ Indeed, thalamic dysfunction that may arise from SARS-CoV-2 infection could be responsible or contribute to a large number of diseases and complications diagnosed in COVID-19-exposed patients, especially linked to neurologic symptoms.^79^ Therefore, the observed thalamic volume loss may offer a clue about the aetiology of widespread cognitive symptoms in PCS and mild COVID-19. Remarkably, our findings support a recent study that links short-term memory problems and fatigue severity to decreased volumes and aberrant diffusion markers of the ventral anterior and ventral lateral nuclei parts of the thalamus in patients with PCS,^80^ which seems to emphasize the contribution of disease-related structural thalamic changes to cognitive symptoms and executive functioning deficits that can occur with a delay of months after COVID-19 infection.

Several studies have put forwards that COVID-19-related WM brain alterations are not a uniform process in a quantitative aspect. Our findings showed that the presence of multifocal juxtacortical WM abnormalities, hyperintense on axial FLAIR images, was higher in mild COVID-19, compared to HC, assessed an average of 9 months after COVID-19 testing, concordantly with the results of previous studies investigating WM abnormalities in moderate-to-severe COVID-19 patients^81-84^. Aside from pointing to GM volume loss in the thalamus and occipital regions, the increase in the number of hyperintensities and (or) juxtacortical lesions might be linked to the same pathophysiological mechanism underlying central nervous system involvement after SARS-CoV-2 infection. The predilection for the presence of WMHs in key brain sites even after mild-to-moderate SARS-CoV-2 infection could be caused by indirect viral pathogenesis through an immune-mediated mechanism and (or) by a detrimental neuroinflammatory response,^85,86^ which may result in small ischaemic processes, hypoxia injury and inflammation where abundant WM fibre tracts and highly myelinated fibre bundles exist.^87^ Moreover, juxtacortical WM, which is mainly made up of U-fibres rather than long WM tracts, is generally supplied by short vessels at the boundary of the WM and cortex, which would be easily influenced by brain microvascular damage and brain inflammation, and that represent some of the main COVID-19-related pathological consequences. Therefore, the finding in mild COVID-19 patients of juxtacortical WM lesions alone may also be symptomatic and might be an early stage of contributing factors that often accompany neurovascular pathology in COVID-19 that subsequently underlie the neurological impact in more severe forms following SARS-CoV-2 infection. In this regard, the elevated juxtacortical hyperintensities observed in our mild COVID-19 sample differ from prior radiological results (i.e. deep and periventricular hyperintensities) identified in PCS patients or a more severe condition. Notably, current evidence indicates that GM and WM abnormalities are remarkable because they seem to be unrelated and independent of the severity of the COVID-19 condition or cognitive function decline being reversible over time. Thus, this could suggest that there are no persistent viral-induced brain abnormalities after mild SARS-CoV-2 infection, but the mechanism of persistent GM and WM dynamic changes in this population, particularly in severe COVID-19, may be triggered by other causes, such as acute hypoxic–ischaemic changes.^81,85,87^

Some potential limitations should be considered when interpreting the results of this study. First, although the experimental groups were matched regarding their demographic characteristics, the small sample size may limit the robustness and generalizability of these results. Therefore, the results reported and conclusions drawn from our study should be interpreted with caution. Second, even though we considered that the 1.5 T scanner met the imaging requirements for our research objectives, it is essential to recognize that higher field strengths are typically expected to afford higher spatial resolution and signal-to-noise ratios. Third, due to the widespread confinement and increased workload experienced by healthcare workers associated with the original COVID-19 epidemic, comparing pre-pandemic HCs with post-COVID-19 patients measured during the pandemic raises challenges regarding anxiety level assessment. We did note, nevertheless, that there are no statistically significant variations in the STAI questionnaire ratings between the groups. Fourth, the lack of a longitudinal study does not allow any prognostic statements regarding the clinical relevance of post-COVID-19 cognitive and brain abnormalities. On the contrary, despite this limitation, our cross-sectional study benefited from the inclusion only of non-hospitalized individuals who developed mild COVID-19 symptoms during the occurrence of SARS-CoV-2 variants Alpha to Delta and also from the fact that the sample was tested in the post-COVID-19 stage at an average of 9 months post-mild infection. This recruitment allowed us to account for chronic cognitive consequences and long-term changes in structural and functional network integrity while avoiding assessing the patients during or near the acute phase, where it would have been easier to detect potentially altered cognitive functioning and acute brain changes associated with mild infection. Fifth, the studied sample was mainly composed of mild COVID-19 cases in healthcare workers, including healthy participants assessed before the pandemic onset. In so, a current limitation is that despite our results that evidence that the exact characterization of potential brain-related abnormalities following mild COVID-19 infection is, however, essential for identifying individuals at higher risk of developing cognitive or structural and functional brain side effects, an open question remains regarding long-term effects, as well as their particular alterations in young or other segments of the population during the original stream. Besides, we did not have any cognitive evaluation of healthcare workers before COVID-19 that could show small differences from baseline in patients but provide compelling evidence of a specific pattern of cognitive impairment and how some brain regions and functional networks are critically affected and reorganized during mild COVID-19 infection. Our results can provide an update on the spectrum of cognitive and neurological manifestations in COVID-19, which may allow the establishment of a common framework easily comparable with other COVID-19 disease courses and translate into studies including other patient populations.

In conclusion, in this study, we report that, when compared to an HC group tested before the COVID-19 pandemic, non-hospitalized mild COVID-19 displayed impaired executive functioning, weaker functional connectivity, thalamic and occipital volume loss and increased juxtacortical WM hyperintensities. Given that these discrete cognitive, functional and structural abnormalities have been similarly seen in several COVID-19 patients and could not be attributed to any other pathology or condition, a connection to COVID-19 seems and, therefore, plausible. Although current evidence suggests that most of these brain alterations in mild COVID-19 are reversible and exhibit consistent recovery over a middle/long period of time, further longitudinal follow-up studies are needed to confirm this speculation. Together, all the functional and structural findings observed in our study point to multiple factors involving dysregulated peripheral immune system activation and subsequent neuroinflammation, coagulopathy and endothelial dysfunction, which may critically impact the brain’s morphology and functions through indirect pathways^15,83,88^ influencing different ways the mental health, cognitive and clinical patterns exhibited by COVID-19 survivors. Our findings also claim the need for routine neuropsychological assessments in all patients infected with SARS-CoV-2 to manage potential cognitive complaints and to unmask and prevent subtle cognitive deficits. Though our findings are far from conclusive, they are startling in their scope and magnitude, spanning new evidence to the neurological repercussions of mild COVID-19 despite the short-term recovery, and raise the question of the impact of COVID-19 on the health status of middle-age healthcare professionals^18,23,24^ during the more dangerous original strain.

## Data Availability

The data supporting the findings of this study, without information that could compromise the privacy of research participants, are available from the corresponding author upon reasonable request.
